# Supplementary material for: Development of a Monoclonal Antibody and a Serodiagnostic Lateral-Flow Device Specific to Rhizopus arrhizus (Syn. R. oryzae), the Principal Global Agent of Mucormycosis in Humans
Source: J Fungi (Basel). 2022 Jul 21;8(7):756. doi: 10.3390/jof8070756 (PMC9325280; doi:10.3390/jof8070756)
Supplement: Supplementary file 1 [file jof-08-00756-s001.zip › jof-1752777-supplementary/jof-1752777-supplementary.pdf]

Davies & Thornton Supplementary Figures.

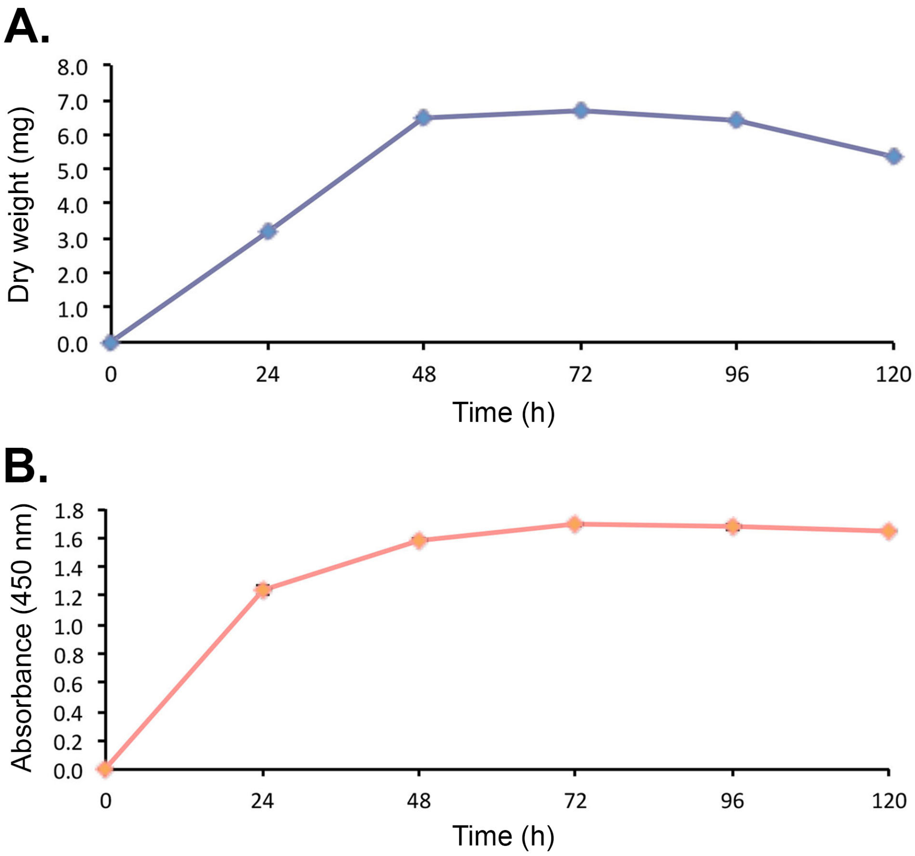

Figure S1. Production of the KC9 antigen by *Rhizopus arrhizus* var. *arrhizus* CBS112.07 grown at 37 ºC in YNB+G shake culture. (**A**) Dry weights of the pathogen over the 5-day experimental period. (**B)** Direct ELISA of culture filtrates using mAb KC9. Each data point (**A, B**) is the mean of 2 replicates ± SE, and the threshold absorbance value for detection of antigen in ELISA (**B**) is ≥0.100.

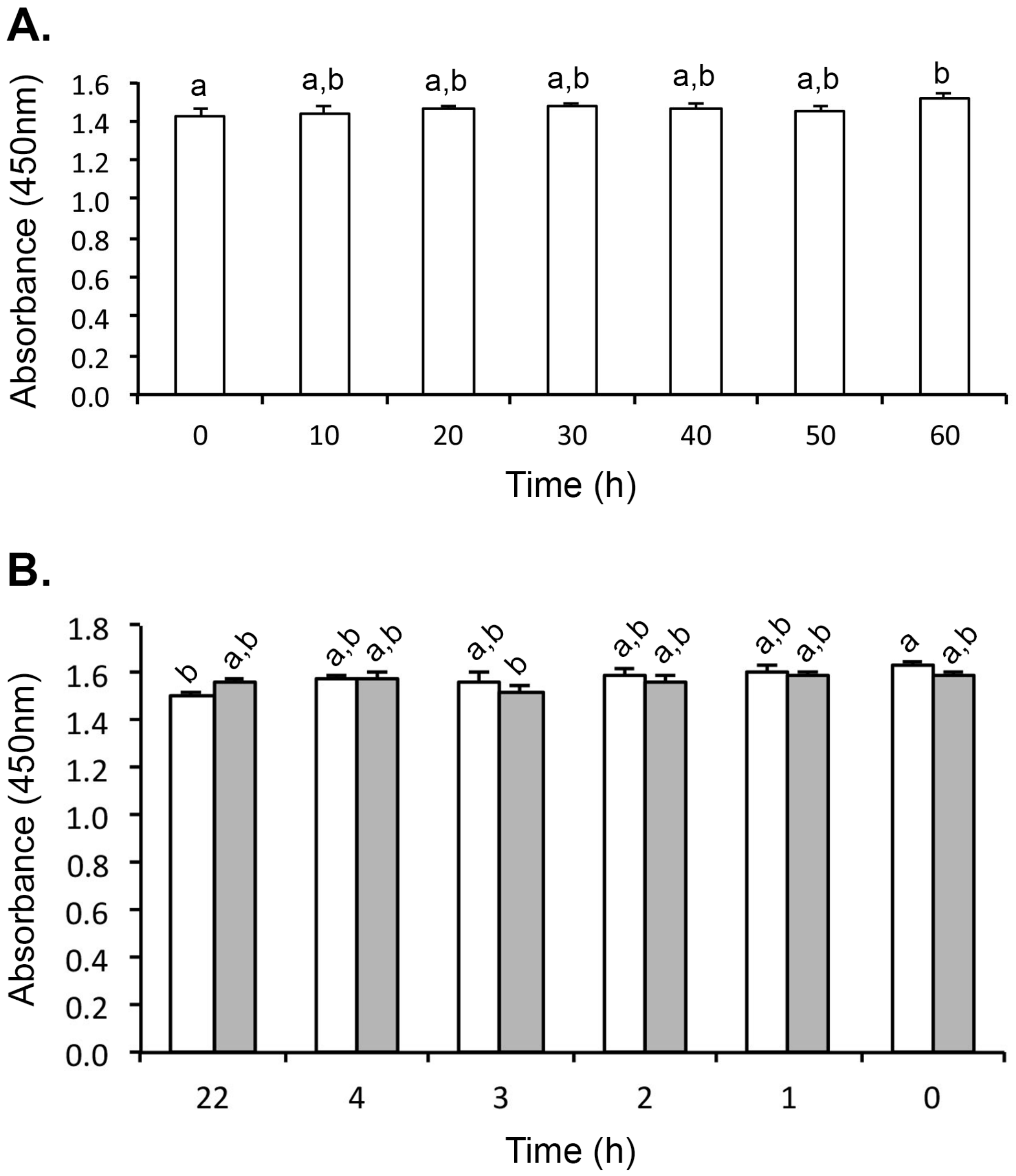

Figure S2. Heat and periodate stability of the KC9 epitope. (**A**) Effect of heat treatment on binding of mAb KC9 to EPS from *R. arrhizus* var. *arrhizus* strain CBS112.07. There was no significant effect on mAb binding over the 60 min period of heat treatment. (**B**) Effect of periodate oxidation on mAb binding to EPS from *R. arrhizus* var. *arrhizus* strain CBS112.07. There was no significant effect of periodate treatment (shaded bars) compared to the control (open bars) over the 22 h period of treatment. For both treatments, bars are the means of 3 replicates ± SE, and bars with the same letters are not significantly different at $p < 0.05$.
